# Restoring trust in truth-seekers: Effects of op/eds defending journalism and justice

**DOI:** 10.1371/journal.pone.0251284

**Published:** 2021-05-21

**Authors:** Raymond J. Pingree, Martina Santia, Kirill Bryanov, Brian K. Watson

**Affiliations:** 1 Manship School of Mass Communication, Louisiana State University, Baton Rouge, Louisiana, United States of America; 2 The Social and Cognitive Informatics Laboratory, Higher School of Economics, St. Petersburg, Russia; Georgia State University, UNITED STATES

## Abstract

A healthy democracy requires trust that people can be impartial in important truth-seeking institutions including journalism, justice, and science. Recently some U.S. elites have adopted alarmingly extreme rhetoric against truth-seekers, denouncing mainstream journalism as fake news, criminal investigations as partisan witch-hunts, climate science as a hoax, and career civil servants as a deep state conspiracy. In response, some news organizations have taken the unusual step of publishing op/eds defending these institutions. Two experiments tested effects of such op/eds. In study 1, participants spent twelve days using a purpose-built news portal containing real, timely news with random assignment to the availability of real, timely op/eds defending impartiality of truth-seekers. These op/eds increased trust in truth-seeking institutions and increased the belief that people can serve as impartial professionals. Study 2 replicated this with a laboratory experiment assigning video op/ed exposure instead of text op/ed availability while adding several outcomes.

## Introduction

If people believe that all information is partisan propaganda, politics becomes a sport with no referees, leaving little hope for meaningful accountability, evidence-based policy, or compromise. Healthy democracy requires a basic form of trust in various kinds of referees of facts relevant to politics, similar to the trust required for actual referees in sports: not a blind faith that they never make mistakes, but trust that they are trying to call the game accurately regardless of which side it helps. Contrary to widespread cynical rhetoric, there are an abundance of referees worthy of this basic trust, not only in the broad truth-seeking institutions of journalism, the justice system, and science, but also in various specific organizations with important truth-seeking roles within and beyond government. Noteworthy examples in the U.S. context include the Congressional Budget Office, the Federal Reserve, the Census Bureau, and the Centers for Disease Control.

This is an unusual historical moment in which impartiality of truth-seeking professions is a matter of explicit and prominent two-sided public debate. Prominent U.S. elites have adopted alarmingly extreme rhetoric against truth-seekers, including referring to mainstream journalism as “fake news” and “the enemy of the people” [1], climate science as a hoax [2], a justice department criminal investigation as a partisan witch hunt [3], and various career public servants within government agencies as a “deep state” conspiracy against the President [4].

The extremity of this rhetoric appears to have provoked an unusual willingness among various kinds of truth-seeking professionals to speak publicly in defense of their professions, which is often seen as contrary to the norms of these professions. Many news organizations have taken the unusual step of publishing op/eds defending the impartiality of journalism [5], and similarly have defended the impartiality of the Justice Department and the Trump-Russia investigation (see for example, [6, 7]). Commentary about an institution may be more influential on trust in that institution than anything the institution actually does, as evidenced by research on effects of anti-media rhetoric [8, 9] and by one recent experiment on effects of rhetoric defending journalism [10]. This suggests that the present moment of public debate about truth-seeking institutions may prove to be a turning point in public trust in those institutions and belief that it is possible for people to be politically impartial professionals.

This study used two experiments, a field experiment embedded in a purpose-built online news portal and a lab experiment embedded in an online survey, to test the effects of exposure to opinion/editorial stories defending impartial professionalism of journalism and of the Justice Department / Trump-Russia investigation. In study 1, participants were paid to spend twelve days using our news portal, which contained real, timely news stories from many sources. Half of participants were randomly assigned to encounter a small number of experimental treatment editorials within the news feed, but all participants were free to make their own choices about which stories to click on to read in more detail. This method aims to maximize realism by using timely content and choice-driven news use. Study 2 is a more traditional lab experiment that directly assigns exposure instead of availability of treatment stories, uses video instead of text treatments, and includes additional outcome measures.

### Attacking and defending journalism

As noted above, the present seems an unusual historical moment in which there is prominent two-sided public debate about the impartiality of a wide variety of truth-seekers. However, one subset of this–rhetoric questioning journalistic impartiality–has a long history and has been well studied. Research on the effects of media bias accusations has important lessons that may inform predictions about effects of defending journalism, as well as effects of rhetoric attacking or defending impartiality of other truth-oriented institutions.

First, anti-media rhetoric has been quite influential [8, 9, 11, 12]. In a time-series study, Watts and colleagues examined some of the factors that contributed to rising public perception of liberal bias in news [9]. This study combined public opinion data on media bias perception, content analysis of actual news favorability to each party, and content analysis of accusations of liberal bias during the 1988, 1992, and 1996 presidential elections. The researchers found that actual press favorability toward each party had no relationship with changes in perceived media bias, but that accusations of liberal media bias did. The same pattern of results has been found in experiments: accusations of media bias reduced media trust, but favorability of news coverage toward each party had no effect [8].

Second, unwillingness to respond in defense of one’s own profession is a normal response in truth-oriented professions, but one that can make the situation worse. Until recently, most journalists took the position that journalists should not respond to attacks because such a response would smack of advocacy, and because ignoring attacks was assumed to be the best way to prove their own neutrality and thus prove the critics wrong [13]. This is an admirably principled stance, but it rests on an unsupported empirical assumption about effects on the audience. After three decades of largely unanswered accusations of liberal bias and declining media trust, it seems to be finally dawning on journalists that the public does not assume their silence on the question of their own bias is evidence of their own principled neutrality. Instead, when people hear an accusation many times from one side and no rebuttal from the accused, people eventually assume the point is conceded. This is a well understood phenomenon in persuasion research: imbalanced flows of arguments can be strongly persuasive over time, even if individual messages have little to no effect [14]. One recent experiment has tested effects of defending journalism and found that the combination of defending journalism and fact checking increases media trust and does so regardless of party identity [10].

Accusations of bias from prominent political elites are nothing new. Rhetoric about liberal mainstream media has roots in the 1970s and 1980s, when the highly charged political atmosphere raised questions about the objectivity of mainstream news organizations and other key U.S. institutions. As journalism underwent a process of professionalization, complaints about a liberal bias of the media became common [9]. During the Nixon administration, for instance, Spiro Agnew led the right’s campaign referring to the media as the “small unelected elite” leaning towards a liberal direction [15]. This anti-media rhetoric seems to have become more extreme in recent years. Since 2016, mainstream news organizations have repeatedly been accused of being “fake news” and “the enemy of the people” [1]. This is a dramatic escalation from mere liberal bias. The word bias at least in its literal meaning denotes an unintentional tendency, whereas much of current anti-media rhetoric is essentially a conspiracy theory that mainstream journalists are intentional partisan operatives.

In response to this escalating anti-media rhetoric, journalists have started to defend their profession and explicitly argue that they can act impartially in verifying facts. Perhaps the most prominent example of this, although by no means the first, is an editorial campaign organized by *The Boston Globe* in August 2018. This campaign led to over 300 American newspapers to publish editorials defending journalism [5]. In an effort to denounce what has been called a “dirty war against the free press,” editorial boards agreed to participate in this initiative in order to restore public faith in their profession and defend the practice of journalism [5].

### Media trust

First among our outcome variables is media trust, defined as a set of closely related evaluations of the media such as fairness, accuracy, telling the whole story, and being unbiased [16]. Although people do also have specific attitudes about specific media outlets that they either use often or hear about often, generalized trust in the mainstream media has been well validated as a stable and consequential attitude among members of the U.S. public [11]. General media trust declined steadily from 1972, when 72% said they trust the media a great deal or a fair amount, until 2016, when that figure reached a low of 32% before rebounding to 45% by 2018 [17]. It seems to us that the timing of this rebound coincides neatly with when journalists became much more willing to speak out in defense of their profession in response to extreme anti-media rhetoric, as discussed above. Future research should test this intuition with a systematic content analysis.

### Political impartiality of justice

In the U.S. context, impartiality of the justice system has until recently been most commonly discussed in terms of race, with concerns raised generally by liberals about unequal treatment of racial minorities and conservatives generally taking the opposite side of defending law enforcement [18]. Recently, in response to the Trump-Russia investigation of a Republican president, this pattern has dramatically shifted both in terms of which side is questioning impartiality and in terms of the nature of the bias accusations shifting from racial bias to partisan bias. Because of these two very different meanings of impartiality of the justice system, we use a novel outcome that focuses on perceived political impartiality of the justice system when investigating politicians.

### Possibility of impartial professionalism

Defense of impartiality of specific institutions may have more general effects on beliefs about people in general, beyond those institutions. Specifically, we will focus on a general belief we label possibility of impartial professionalism (PIP). PIP is the belief that it is possible for people to set aside their own political biases and make fair evidence-based judgments about facts. PIP is conceptually distinct from trust in specific truth-oriented institutions because PIP focuses only on individuals and their ability to set aside their own preferences. Thus, it may be a common foundation for trust in each of these institutions. Without some degree of belief that individuals can act as impartial truth-seeking professionals, people are unlikely to trust these institutions. Further, from the perspective of anyone looking for ways to restore trust in these important institutions, PIP seems a more tractable first step. In other words, it seems to us that it would be relatively easy to convince people that it is at least possible for human beings to serve as relatively impartial truth-seekers, even if they still believe, for example, that most mainstream journalists are partisan actors instead of impartial truth-seekers.

## Hypotheses and research question

As reviewed above, past research suggests that exposure to messages defending the impartiality of truth-seeking institutions may increase trust in these institutions. Here the institutions we focus on are news media and the justice system. In the case of the justice system, we focus specifically on trust in its ability to be politically impartial when investigating politicians, an attitude we label as “justice political impartiality” or JPI. We also test whether defense of these two truth-seeking institutions affects more general attitudes beyond attitudes about the specific institutions defended in the messages. Specifically, both study 1 and study 2 also test a general belief that it is possible for people to act as impartial, truth-seeking professionals, an attitude we label as “possibility of impartial professionalism” or PIP. In sum, both studies test the following main hypothesis:

*H1*: *Defense of impartiality will increase a*.*) mainstream news trust*, *b*.*) justice political impartiality*, *and c*.*) belief in the possibility of impartial professionalism*.

Two additional outcomes are added in study 2. First, we test effects on perceived political impartiality of several other kinds of truth-seekers beyond justice and news media (i.e., public opinion polling organizations, federal judges, the Federal Reserve, and the Congressional Budget Office). Second, study 2 also examines whether effects of defense of impartiality go beyond abstract attitudes about truth-seekers and also apply to specific outputs of their truth-seeking. Specifically, we test whether defense of journalism affects the perceived accuracy and importance of two timely political scandal news stories published by major news outlets immediately prior to the experiment. In sum, study 2 adds the following predictions about effects of defending impartiality:

*H2*: *Defense of impartiality will increase a*.*) the perceived impartiality of other truth-seekers and b*.*) evaluations of political scandal news stories*.

Partisan preference may condition any of the above effects. Republicans may be particularly resistant to defense of impartiality due to recent rhetoric from some Republican elites against various truth-seeking professions. On the other hand, past exposure to defense of impartiality may differ across party lines, so effects of defense of impartiality could also perhaps be more influential on Republicans than Democrats due to the relative novelty of the stimulus. Thus, we also pose a broad research question about the moderating role of partisanship.

*RQ*: *Will party preference moderate any of these effects*?

## Study 1 methods

**Fig 1:**
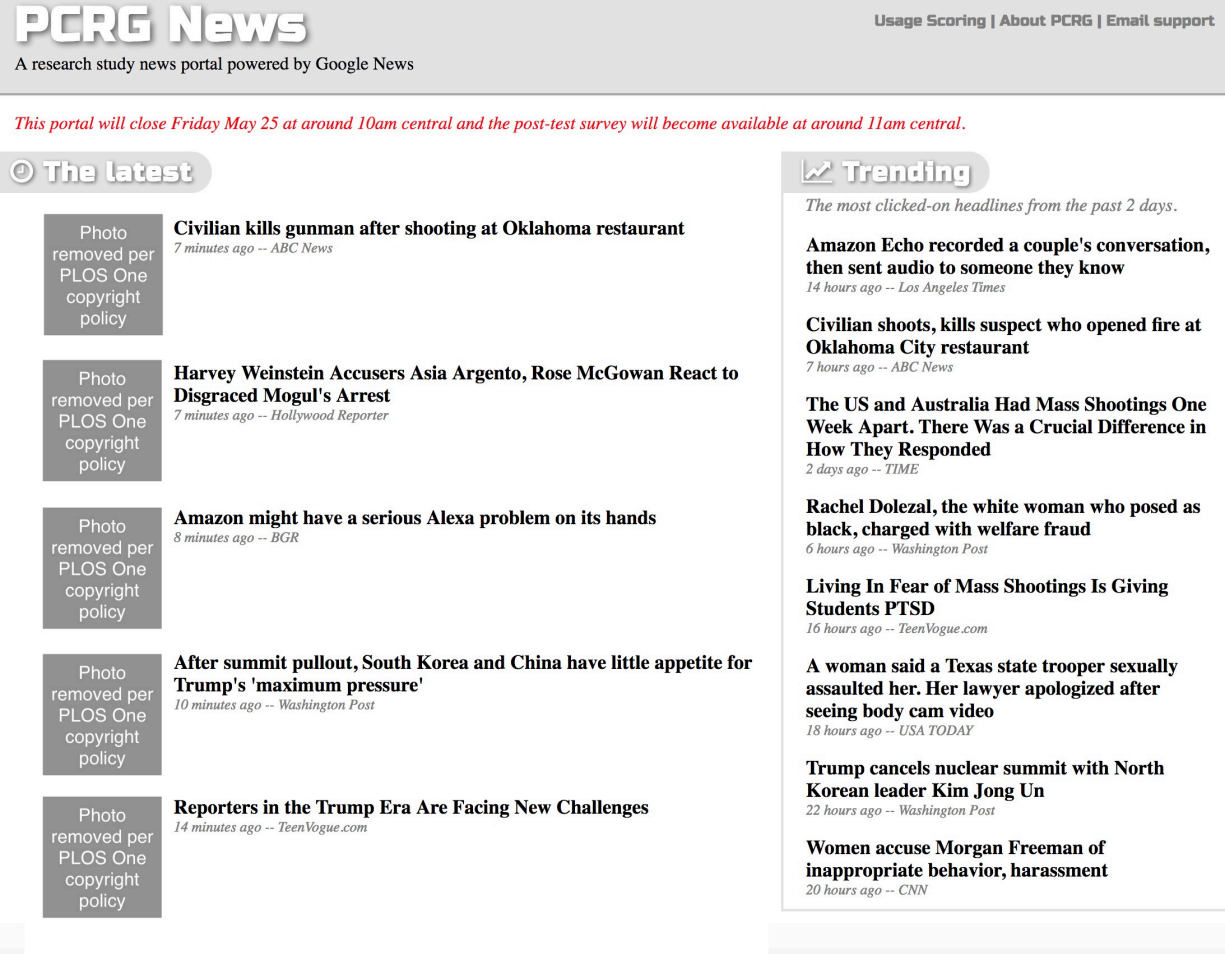
Study 1 news portal screenshot.

Study 1 used a purpose-built online news portal website (browser-based and also mobile-accessible) similar to Google News to manipulate the availability of defense of impartiality stories during choice-driven exposure to real, timely news content (Fig 1). The customized portal automatically added timely news stories from a variety of verified news outlets (both mainstream and partisan) at the top of every hour for twelve days in May 2018. Newly found stories did not appear in the portal for at least 5 minutes, allowing researchers on two continents to verify keyword-based categorization of stimulus stories before they appeared. By the end of the twelve-day period, the portal included a total of 3189 news stories. We use a single two-level treatment factor (defense of impartiality stories added or not), drawn from a fully factorial design intended for multiple other purposes beyond the scope of this paper. Non-hypothesized factors are included for control purposes in all analyses. This study was approved by LSU IRB April 26, 2018 (IRB #E11057).

### Participants and procedures

A convenience sample of U.S. adults (final N = 1391; 54.9% female, 44.7% male, .4% other) was recruited through Amazon’s Mechanical Turk (MTurk), an online service allowing workers to participate in studies for a nominal payment. Participants were required to be U.S. residents, 42.3% had a four-year college degree, with an average age of 39 years (SD = 12.28). Participants were 77.5% Caucasians, 7.4% African Americans, 7.1% Asians, 6.0% Hispanics, .1% Pacific Islanders, and 2.0% indicated other or multiple ethnicities. 26.0% identified as Republicans, 47.8% Democrats, 22.8% Independents, and 3.4% other.

Study participants were instructed to use the custom-built newsfeed and consume news like they would in a normal web-browsing session. Participants were free to choose which stories to read in detail. When they clicked on any story of interest, they were redirected to the website from which the story was published. Each participant encountered an average of 493 headlines and clicked on 55 of them to read in detail. Participants were paid $1 for the pre-test survey, $1 for the post-test survey (which became available an hour after the portal closed), and between $1 and $3 based on the amount of time actually spent in the portal. Usage incentives were intended to be insufficient to motivate substantially increased news use. As explained to users, the modest payment was intended as compensation for using our news portal instead of other news sources but was not intended to compensate them for spending more time with news than they otherwise would. Although we do not have measures of time spent reading news or any other behavior that occurred on external websites where the actual news stories were read, the mere fact that participants clicked on an average of 55 stories suggests that usage was far higher than one would expect in a traditional forced exposure lab experiment paying $3. This supports our view that the primary reason participants used the portal was that it contained news stories they wanted to read. Thus, embedding experimental treatments within an actually useful news portal has the advantage of producing a more externally valid motivational context while efficiently producing high stimulus exposure at relatively low cost per participant.

### Defense of impartiality treatment

At the top of each hour, automatic RSS queries pulled stories from Google News matching a set of keywords related to journalism as well as to the FBI / Trump-Russia investigation. Candidate stories for the defense of impartiality treatment were found through keywords related to journalism, the FBI and/or the Department of Justice (DOJ), and the Trump-Russia investigation. Examples of stories defending the Trump-Russia investigation were “Here’s what the special counsel’s Trump-Russia probe has accomplished after one year” and “Ruth Marcus: Mueller is conducting no Witch Hunt.” Examples of stories defending the FBI/Department of Justice were “DOJ quickly responded after Trump said he’ll demand an investigation into whether politics were behind…” and “Rosenstein extols DOJ’s embrace of ethics as Trump derides Russia witch hunt.” Stories defending the integrity of journalism included “Spencer Black: fake news = any news Donald Trump doesn’t like” and “Bill Knight: negative news doesn’t equal fake news.” None of these candidate stories were automatically published in the portal. The researchers monitored the portal 24 hours a day and carefully verified categorization of stories and selected which ones were about defense of impartiality before adding them to the portal.

Timely news stories were preferred over non-timely ones. However, when there were not enough available, the researchers manually added older stories to ensure publication of at least three treatment stories a day. The oldest stories added to the portal were published just a few days before the portal went live (May 8, 2018). At the end of the twelve-day period, the portal featured a total of 58 stories defending impartiality. On average, participants in the defense of impartiality condition saw 5.05 headlines defending impartiality (SD = 9.94) and clicked on .54 of them (SD = 1.88). Of these stories, eight defended the FBI and/or the DOJ, 17 defended the integrity of the Mueller probe, and 33 defended journalism (for a full list of published stories, see S1 Appendix).

### Measures

All outcome variables were measured on a seven-point Likert-type scale ranging from “1” labeled as “strongly disagree” to “7” labeled as “strongly agree.”

Mainstream news trust (M = 3.48, SD = 1.58, α = .96) was an average of five items from a scale developed by Gaziano and McGrath [16] and later refined by Tsfati [19]. Participants were asked whether they agreed or disagreed with a set of closely related evaluations of mainstream news outlets. The first item was, “in general, mainstream news outlets are fair.” The other four statements used the same exact wording but replaced “are fair” with “are accurate,” “are unbiased,” “tell the whole story,” and “can be trusted.”

Justice political impartiality (JPI) was an average of two novel items (M = 4.81, SD = 1.77, r = .73): “high ranking people in the FBI, Department of Justice, and the Mueller probe are biased against the President” (reverse-coded) and “high ranking people in the FBI, Department of Justice, and the Mueller probe are professionals just trying to get the facts.”

Belief in the possibility of impartial professionalism was a single item: “it is possible for people to be truth-seeking professionals, meaning they do their jobs fairly regardless of which political party it helps or hurts” (M = 5.64, SD = 1.39).

## Study 1 results

Hypothesis 1 predicted that defense of impartiality will affect three outcomes: a) mainstream news trust, b) justice political impartiality, and c) belief in the possibility of impartial professionalism. To test this hypothesis and our research question, we used three analysis of covariance (ANCOVA) models, one for each outcome variable. Each model included the pre-test version of each outcome variable as a covariate. These covariates were necessary to guard against the possible confound of systematic differences in attrition across experimental conditions. Other than their dependent variables and covariates, the three ANCOVA models were identical, each consisting of: a two-level factor for the defense of impartiality manipulation, a three-level party preference factor (Republican, Democrat, or Independent/Other), other non-hypothesized experimental factors as main effects, and a two-way interaction between defense of impartiality and party preference.

In the model predicting mainstream news trust (adjusted R^2^ = .763), the defense of impartiality main effect was significant (F[1, 1366] = 2.867, one-tailed *p* = .045), and in the predicted direction, with a higher mean (M = 3.464, SE = .039) in the defense of impartiality condition than in the control condition (M = 3.371, SE = .039), so H1a was supported. In answer to the RQ, these effects were significantly conditioned by partisanship (F[2, 1366] = 3.356, two-tailed *p* = .035) with particularly strong effects among Republicans and Independents.

In the model predicting justice political impartiality (adjusted R^2^ = .762), the defense of impartiality main effect was also significant (F[1, 1338] = 8.600, two-tailed *p* = .003), and in the predicted direction, with a higher mean (M = 4.804, SE = .044) in the defense of impartiality condition than in the control condition (M = 4.622, SE = .044), so H1b was also supported. In answer to RQ, partisanship did not moderate these effects (F[2, 1338] = .790, two-tailed *p* = .454).

The model predicting the belief in the possibility of impartial professionalism (adjusted R^2^ = .348) supported H1c. The defense of impartiality main effect was significant (F[1, 1351] = 10.401, two-tailed *p* = .001), and in the predicted direction, with a higher mean (M = 5.655, SE = .056) in the defense of impartiality condition than in the control condition (M = 5.399, SE = .056). In answer to RQ, these effects were significantly conditioned by partisanship (F[2, 1351] = 4.303, two-tailed *p* = .014), again with particularly strong effects among Republicans and Independents. For a summary of results see Table 1 below.

**Table 1:** Study 1 treatment mean differences.

	Estimated marginal means		
	Treatment	Control	Treatment—control
**Mainstream news trust**	3.464	3.372	.092*
Democrats	3.553	3.622	-.069
Independents	3.493	3.221	.272
Republicans	3.346	3.273	.073
**Justice political impartiality**	4.804	4.622	.182**
**Possibility of impartial professionalism**	5.655	5.399	.256***
Democrats	5.819	5.830	-.011
Independents	5.692	5.131	.561
Republicans	5.453	5.237	.216

Note:

*p < .05

**p < .01

***p < .001. Party splits included only where party significantly interacted with the treatment.

## Study 2 methods

Study 2 uses an experiment embedded in an online survey that manipulated exposure to a treatment video defending journalism and two control videos. In contrast to study 1, which manipulated availability of timely stimuli but allowed participants to choose which full stories to view, study 2 is a more traditional laboratory experiment that directly manipulates exposure to stimuli. A three-level video treatment factor is used here (defense of impartiality video, immigration control video, movie trailer control video), drawn from a larger fully factorial design intended for other purposes beyond the scope of this study. Non-hypothesized factors are included for control purposes in all analyses. This study was approved by LSU IRB January 17, 2019 (IRB #E11438).

### Participants and procedures

As for study 1, we recruited a convenience sample of U.S. adults (final N = 1364; 50.4% female, 49.4% male, .2% other) from Amazon’s Mechanical Turk online survey service for study 2. Participants were U.S. residents, 42.8% had a four-year college degree, with an average age of 39 years (SD = 12.81). Participants were 74.3% Caucasians, 9.2% African Americans, 7.2% Asians, 6.6% Hispanic and/or Latinos, .2% Pacific Islanders, and 2.5% that indicated other or multiple ethnicities. 55.9% of the sample indicated a preference for the Democratic Party, 30.9% preferred the Republican Party, and 13.1% did not disclose any party preference. Participants received $1 compensation for completing the study.

### Stimulus videos

Participants randomly assigned to the defense of impartiality condition saw a two-minute (2:04) video clip consisting of two segments. The first segment was from Fox News Chief Anchor Shepard Smith titled “Journalists are not the enemy of the people” originally published on July 26, 2018. The second segment was a clip from a panel discussion in the PBS News Hour from January 23, 2018 in which New York Times columnist David Brooks defended the professional impartiality of the FBI and beyond, saying that “there are career people who really do their job, and they try to be good umpires.” He then added that in Washington “there are a lot of agencies that are filled with honest brokers” who “are not actually that political” but instead “believe in the public service and then try to do their jobs” (see S2 Appendix for transcripts of the videos).

Two different kinds of control videos were used. First was a two-minute (2:14) movie trailer for an upcoming action movie commonly being advertised at the time (Captain Marvel), intended to be unrelated to any of the content of the study. The other control was an immigration video that was intended to be as similar as possible to the treatment video but without including defense of impartiality. Thus, following the format of the treatment video, the immigration control video was a two-minute (2:01) video with two segments from the same two news outlets and speakers as the first video. In this video, both Smith and Brooks criticized President Trump’s rhetoric about an immigrant caravan. In particular, this criticism from Fox News host Shephard Smith was thought to be important for this control condition in terms of isolating our intended defense of impartiality treatment from the confound of expectancy violation. The defense of impartiality treatment video could be seen as surprising criticism of a Republican president from Fox News, an outlet normally thought of as favorable to Republicans. Thus, this control condition aimed to create this same expectancy violation by showing a different criticism of Trump from the same Fox News host, without any mention of professional impartiality.

### Measures

Mainstream news trust (M = 3.66, SD = 1.62, α = .96) was an average of the same 5 items from study 1.

Justice political impartiality (JPI) was expanded from the two-item scale used in study 1 to a five-item scale (M = 4.51, SD = 1.58, α = .96). The first item was, “when investigating politicians, the FBI and Justice Department are fair.” The subsequent four statements used the same wording, replacing “are fair” with “are driven by evidence,” “are non-partisan,” “are not biased by private political beliefs of their employees,” and “can be trusted.” The seven-point scale ranged from “1” labeled as “strongly disagree” to “7” labeled as “strongly agree.”

We also expanded our single-item measure of belief in the possibility of impartial professionalism from study 1 to a five-item scale (M = 5.47, SD = 1.26, α = .94) including: “it is possible for people to be truth-seeking professionals, meaning they do their jobs fairly regardless of which political party it helps or hurts;” “it is possible for people to set aside their political opinions and seek the truth;” “it is possible for people to be impartial when dealing with highly politicized issues;” “it is possible for people to reach unbiased conclusions about political facts;” and “it is possible for people to rely on evidence rather than politics when seeking the truth.” Responses ranged from “1” labeled as “strongly disagree” to “7” labeled as “strongly agree.”

We measured perceived impartiality of several other kinds of truth-seekers each with a single item and elected to combine them into a four-item scale due to relatively high reliability (M = 6.40, SD = 1.80, α = .82). Items asked about the impartiality of “Public opinion polling organizations,” “Federal judges,” “the Federal Reserve (or Fed, in charge of setting interest rates to stimulate or cool off the economy),” and “the Congressional Budget Office (or CBO, in charge of predicting how each bill will affect the budget).” These items were grouped under a heading “For each of the following, how often do you think they set aside personal political views and make fair decisions based on evidence?” Response options for each item ranged from “1” labeled as “never” to “10” labeled as “always.”

Finally, we measured evaluations of political scandal news stories (M = 4.97, SD = 1.74, α = .93) by averaging respondents’ attitudes toward two recent news stories about political scandals involving President Trump. Specifically, one story dealt with an FBI inquiry into whether President Trump was secretly working on behalf of Russia and the other story was about concealed details of President Trump’s recent face-to-face encounter with Putin. The first story had been published by the New York Times while the second had been published by the Washington Post, both just a few days before the experiment was launched. To create a scale for evaluations of political scandal news stories, we averaged answers to two questions concerning attitudes toward the two news stories. The first question asked participants how likely it was that the story was accurate with response options ranging from “1” labeled “not at all likely” to “7” labeled “very likely.” The second question asked participants how important they thought it was for the public to read, with response options ranging from “1” labeled “not at all” to “7” meaning “extremely important.”

## Study 2 results

For Hypothesis 1, we predicted that defense of impartiality will increase three outcomes: a) mainstream news trust, b) justice political impartiality, and c) belief in the possibility of impartial professionalism. To test the effects of defense of impartiality, we used three ANCOVA models, one for each outcome variable. Next, we constructed a dichotomous variable to isolate the three video conditions more efficiently. Each ANCOVA model included: a dummy variable for the immigration control video, a dummy variable for the movie trailer control video, a three-level party preference factor (Democratic Party, Republican Party, or no party preference), and two other non-hypothesized experimental factors (yielding no significant effects). The defense of impartiality condition was the missing dummy and served to compare the effects of defending impartiality to the other two conditions. We included several control variables in our models to guard against the possibility of differential dropout from the study. We asked participants what their highest level of formal education was and how interested they were in politics, which was assessed on a seven-point scale ranging from “1” meaning “not at all” to “7” meaning “very interested.” The model also included feeling thermometers as covariates, which asked respondents to rate how warmly they felt toward President Trump and towards mainstream news media.

The ANCOVA model predicting mainstream news trust (adjusted R^2^ = .638) revealed a significant main effect for the defense of impartiality video as opposed to both the immigration video (F[1, 1304] = 14.725, two-tailed *p* < .001) and the movie trailer (F[1, 1304] = 9.299, two-tailed *p* = .002), thus supporting H1a. In response to the RQ, these effects were not moderated by party preference. Both the interaction with the immigration video (F[2, 1304] = .358, two-tailed *p* = .699) and the interaction with the movie trailer (F[2, 1304] = .076, two-tailed *p* = .927) were not significant.

The model predicting justice political impartiality (adjusted R^2^ = .390) revealed a significant main effect for the defense of impartiality video as opposed to both the immigration video (F[1, 1304] = 14.032, one-tailed *p* < .001) and the movie trailer (F[1, 1304] = 3.835, one-tailed *p* = .025), thus supporting H1b. In response to the RQ, these effects were not moderated by party preference. Both the interaction with the immigration video (F[2, 1304] = 2.406, two-tailed *p* = .091) and the interaction with the movie trailer (F[2, 1304] = .633, two-tailed *p* = .531) were not significant.

The model predicting the belief in the possibility of impartial professionalism (adjusted R^2^ = .072) revealed partial support for H1c. While the analysis revealed a significant main effect for the defense of impartiality video as opposed to the immigration video (F[1, 1302] = 3.387, one-tailed *p* = .033), the effect of the defense of impartiality video as opposed to the movie trailer video was not significant (F[1, 1302] = 1.534, one-tailed *p* = .108). In response to the RQ, party preference did not moderate these effects. Both the interaction with the immigration video (F[2, 1302] = .386, two-tailed *p* = .680) and the interaction with the movie trailer (F[2, 1304] = .468, two-tailed *p* = .626) were not significant.

Hypothesis 2 predicted that defense of impartiality will affect two additional outcomes a) the perceived impartiality of other truth-seekers and b) evaluations of political scandal news stories. To test this hypothesis, we used two additional ANCOVA models, one for each outcome variable. Each model also included the same control variables from the ANCOVA models used for H1.

The model predicting perceived impartiality of other truth-seekers, including polling organizations, federal judges, and the Congressional Budget Office (adjusted R^2^ = .281) revealed a significant main effect for the defense of impartiality video as opposed to both the immigration video (F[1, 1272] = 6.159, one-tailed *p* = .006) and the movie trailer (F[1, 1272] = 2.841, one-tailed *p* = .046), thus supporting H2a. To answer the RQ, these effects were not moderated by party preference. Both the interaction with the immigration video (F[2, 1272) = .000, two-tailed *p* = 1.00) and the interaction with the movie trailer (F[2, 1272] = .826, two-tailed *p* = .438) were not significant.

The model predicting evaluations of political scandal news stories (adjusted R^2^ = .573) revealed a significant main effect for the defense of impartiality video as opposed to both the immigration video (F[1, 1307] = 4.657, one-tailed *p* = .015) and the movie trailer (F[1, 1272] = 7.438, one-tailed *p* = .003), thus showing support for H2c. These results show that defending impartiality changes how people evaluate quality of the outputs of truth-seeking institutions in the midst of the Trump-Russia scandal. To answer the RQ, party preference moderated these effects in the non-news video condition (F[2, 1307] = 4.477, two-tailed *p* = .012). We followed this up with separate models for Democrats, Republicans, and Independents, finding that defending impartiality increased Trump-Russia story quality among Republicans (F[1, 398] = 6.087, two-tailed *p* = .014), but did not significantly affect this outcome among others. For a summary of results for study 2, see Table 2 below.

**Table 2:** Study 2 treatment mean differences.

	Estimated marginal means		
	Treatment	Treatment–immigration control	Treatment–movie trailer control
**Mainstream news trust**	3.772	.304***	.239**
**Justice political impartiality**	4.700	.379***	.196*
**Possibility of impartial professionalism**	5.578	.181*	.120
**Other impartiality**	6.530	.317**	.214*
**Evaluation of Trump-Russia stories**	5.073	.200*	.250**
**Democrats**	5.139	.087	-.066
**Independents**	5.165	.158	.444
**Republicans**	4.915	.355*	.372**

Note:

*p < .05

**p < .01

***p < .001. Party splits included only where party significantly interacted with the treatment.

## Discussion

A major understudied threat to democracy is the misperception among citizens that there are no impartial referees to assess any kinds of factual claims related to politics. After several decades of erosion of trust in truth-seeking institutions and in the impartiality of truth-seeking professionals, research is urgently needed to assess the effectiveness of possible interventions to restore this trust. We contribute to this with two straightforward tests of the effects of a traditionally uncommon practice: published editorials defending the impartiality of truth-seeking institutions. In sum, study 1 used a field experiment embedded in an online news portal and found that editorials defending impartiality of journalism and justice increased media trust, justice trust, and the belief in the possibility of impartial professionalism, generally with stronger effects among Republicans and Independents. Study 2 replicated study 1’s findings with a forced exposure lab experiment and found largely similar effects, but without significant differences across parties. Study 2 also added two additional outcomes, perceived impartiality of other truth-seekers beyond journalism and justice, and evaluations of timely political scandal stories (i.e., outputs of truth-seeking institutions), and found that both were significantly increased by defense of impartiality, with the latter effect concentrated among Republicans.

The findings regarding the role of respondent partisanship in conditioning effects were encouraging. A valid concern one might have about this type of intervention is that it may have preached to the choir, only influencing Democrats due to their much higher levels of trust in media [17], or perhaps even to backfiring among Republicans as some fact checks have in past research [20]. This was not what we found for any outcome. Instead, party identity didn’t interact with the treatment for five of the eight interaction tests across the two studies, and for the other three, the treatments were more persuasive to independents in study 1 and among both independents and to Republicans in study 2. In other words, where party mattered, the supposed choir (i.e. Democrats) was actually less persuaded. If this only occurred in study 2, this could have been attributed to the stimulus being from a source less trusted by Democrats (i.e. Fox News), but given that Democrats were also less influenced where partisan differences were found in study 1, the most parsimonious explanation is lower novelty of the stimulus messages among Democrats due to differences in prior media exposure. As with any media effects experiment, even with an unusually long study duration such as in study 1, the treatment is only a drop in the bucket of total relevant media exposure that has shaped each participant’s attitudes for years prior to the study. Thus, partisan differences in experimental effects may be driven not only by differences in persuasiveness of that new drop in the bucket, but also by differences across parties in how many similar drops were already in the bucket.

Study 1 used an innovative method for assessing accumulating media effects over an extended period of time in realistic choice-driven news use. This combines the strengths of a laboratory experiment in allowing a causal test of effects of editorials defending professionalism with the greater realism of observational studies of real news use by employing a much more realistic choice-driven news environment. Instead of measuring editorials’ effects through a conventional single-shot experiment as in study 2, study 1’s method allows to measure the same outcome through repeated exposure to stimuli. Since we used real, timely opinion pieces as experimental stimuli and participants chose which editorial to read, we conclude that the results mirror what occurs in everyday online news use. Because of a minimum one-hour delay after the last possible stimulus exposure and when the post-test survey became available, these effects cannot be interpreted as fleeting and based on temporary cognitive accessibility. Rather, it is reasonable to assume that they are lasting effects and are thus likely to continue to accumulate into larger changes over longer periods of exposure.

The effects found here were not large in absolute terms, but nonetheless were impressive in proportion to the amount of treatment exposure. In study 1, outcome variables measured on seven-point scales were affected by at most about one-quarter of a point, and in study 2 by about a third of a point. Treatment exposure in study 1 was very limited due to allowing participants to choose which stories to click on from a news feed containing many non-treatment headlines and a few treatment headlines. Story content was not visible until a headline was clicked, and the average number of treatment headlines clicked on was about .5. Study 2 may have achieved its slightly larger effects due to slightly higher two-minute stimulus exposure, and by measuring outcomes immediately after exposure.

Since our M-Turk sample is a non-representative sample, the effects may not generalize to the population. This concern is lessened somewhat because we do not aim to accurately estimate population means but instead to estimate changes in means in response to manipulations. Nonetheless, it is important for future research to attempt to replicate these results with a more representative sample. Such a representative sample study would ideally also attempt to further develop and validate measures of professionalism, intended as the belief that others can be politically impartial in key professional roles that intersect politics, including journalism, justice, and science.

Much more research is needed to replicate these results and further develop and validate the outcome measures used here. Complementary methods, such as content analyses or rhetoric about truth-oriented institutions paired with public opinion surveys, similar in methods to Watts and colleagues [9], could directly assess our intuition that prior to 2016 defense of impartiality in journalism was rare, and the rebound in media trust since 2016 correlates with the media’s sudden willingness to respond to anti-media rhetoric. Our possibility of impartial professionalism measure also merits further validation by assessing its relationship with commonly used measures of institutional trust. As we have argued, this may be a valuable measure not only to explain attitudes toward specific truth-seeking institutions, but also as a point of leverage for interventions to restore trust.

The findings suggest that a relatively simple intervention can help restore trust in truth institutions, their outputs, and the professional impartiality of their workers. Considering the recent alarming attacks on truth institutions including journalism, the justice system, and science, it is important to find ways to restore confidence in the belief that professionals can be impartial in important truth-seeking roles that intersect with politics. Our results suggest that journalists can make a difference by simply being more willing to speak up in defense of their own or other truth-oriented institutions.

## Supporting information

S1 Appendix Study 1 stimulus stories. (DOCX)

S2 Appendix Study 2 stimulus video transcripts. (DOCX)
