# Supplementary material for: Restoring trust in truth-seekers: Effects of op/eds defending journalism and justice
Source: PLoS One. 2021 May 21;16(5):e0251284. doi: 10.1371/journal.pone.0251284 (PMC8139466; doi:10.1371/journal.pone.0251284)
Supplement: S1 Appendix — (DOCX) [file pone.0251284.s001.docx]

**S1 Appendix. Study 1 Stimulus Stories.**

| **Source** | **Headline** | |  |
| --- | --- | --- | --- |
| New Republic | Robert Mueller's Most Important Accomplishment | |  |
| Mediaite | FBI Head Christopher Wray: Russia Probe Not a Witch Hunt | |  |
| MyAJC (blog) | Opinion: Mueller is Trump's creation | |  |
| CNBC | Here's what the special counsel's Trump-Russia probe has accomplished after one year | |  |
| CNN | Don Lemon debunks Trump's "witch hunt" claims | |  |
| Akron Beacon Journal | Ruth Marcus: Mueller is conducting no "Witch Hunt" | |  |
| News 13 Orlando | AP FACT CHECK: Trump's tweets on Russia probe short on facts | |  |
| BuzzFeed News | DOJ Quickly Responded After Trump Said He'll "Demand" An Investigation Into Whether Politics Were Behind ... | |  |
| Yahoo News | Rosenstein Extols DOJ's Embrace of Ethics as Trump Derides Russia "Witch Hunt" | |  |
| WRAL.com | Trump's Demands Escalate Pressure on Rosenstein to Preserve Justice Department's Independence | |  |
| The Boston Globe | Trump administration's internal struggle: rule of law or law of ruler | |  |
| Los Angeles Times | Trump can show Mueller's investigation is a witch hunt by releasing his tax returns | |  |
| Daily Beast | Rosenstein Defends Justice Department, FBI From Trump's "Criminal Deep State" Attack | |  |
| Middletown Transcript | Carper stands behind rule of law, Mueller investigation | |  |
| Madison.com | Spencer Black: Fake news = any news Donald Trump doesn't like | |  |
| Observer-Reporter | The sense of justice that we're losing | |  |
| KUNC | From Neil Best's Desk: The Changing Face of Journalism | |  |
| Austin American-Statesman | Commentary: GOP lawmakers defend the president, not the Constitution | |  |
| New York Times | Trump's GOP vs. the Rule of Law | |  |
| WUSA9.com | #OffScriptOn9: Working to Verify what news is real and what news is fake | |  |
| Craig Daily Press | Times change; the mission doesn't | |  |
| WBUR | Only Humans Can Fix Facebook's Fake News Problem | |  |
| The Pew Charitable Trusts (blog) | Finding Facts | |  |
| USA TODAY | Oprah Winfrey condemns fake news in commencement speech, urges graduates to "be the truth" | |  |
| Plattsburgh Press Republican | Fake news is not journalism | |  |
| PBS NewsHour | Sally Yates on Trump's travel ban and protecting the rule of law | |  |
| New Republic | The Scourge of Trumpism in Conservative Journalism | |  |
| Houston Chronicle | We already live in a fake news world - and it's about to get worse. [Opinion] | |  |
| Information Age | Tech vs fake news: Separating the fact from Russian troll farm manipulation | |  |
| Asheville Citizen-Times | EDITORIAL: Mark Meadows' appalling attack on the rule of law | |  |
| Financial Times | Trump, fake news and the seeds of doubt | |  |
| Press Gazette | Newspapers are in a "daily fight against fake news" and IPSO mark shows which side they're on | |  |
| INSCMagazine | Social Media: How To Deal With Fake News and Yellow Journalism | |  |
| ticklethewire.com | Sally Yates: Trump "Tearing Down the Legitimacy" of the Justice Department | |  |
| Mother Jones | Facebook is Enlisting These Disinformation Detection Pros to Fight Fake News | |  |
| Delaware First Media | "A Matter of Facts" podcast: McKay Jenkins | |  |
| Press-Enterprise | The news isn't fake, but it's flawed, New York Times columnist acknowledges at UCR lecture | |  |
| Channel NewsAsia | Commentary: An era of fake news, how the seeds of doubt are sown | |  |
| Voice of America | News Literacy Introduction: News Through Time | |  |
| TheWrap | Hillary Clinton Tells Yale Graduates to Fight Fake News by "Subscribing to a Newspaper" | |  |
| Chicago Sun-Times | EDITORIAL: A president ordering up political investigations attacks rule of law | |  |
| Reuters | DOJ independence, entrenched and ingrained, will survive Trump: historian, law prof | |  |
| Pekin Daily Times | Knight: "Fake news" vs. negative journalism based on facts | |  |
| Slate Magazine | Trump's Assault on Prosecutorial Independence | |  |
| Galesburg Register-Mail | Bill Knight: Negative news doesn't equal "fake news" | |  |
| Social Europe | Fake News And The Fairness Doctrine | |  |
| Washington Post | Trump admitted he attacks press to shield himself from negative coverage, Lesley Stahl says | |  |
| Wall Street Journal | Rod Rosenstein Defends FBI After Trump Attacks | |  |
| Harvard Law School News | Sen. Flake challenges Class of 2018 to protect the rule of law | |  |
| TIME | Read Jeff Flake's Commencement Speech on the Rule of Law and Trump: "We May Have Hit Bottom" | |  |
| RealClearPolitics | Lesley Stahl: Trump Told Me He Uses Term "Fake News" To Discredit The Media | |  |
| Politico | George Conway's Tweets Raise West Wing Eyebrows | |  |
| TeenVogue.com | The "Free Press" Explained: What It Is and How It Works | |  |
| The Atlantic | Elon Musk's Silly War With the Media | |  |
| Fast Company | What Elon Musk and Donald Trump don't get about journalism | |  |
| Charleston Gazette-Mail | HuffPost journalist discusses animosity toward media | |  |
| Arizona Daily Star | Steller's Friday Notebook: Flake defends rule of law at law-school graduation | |  |
| TeenVogue.com | Reporters in the Trump Era Are Facing New Challenges | |  |
|  | |  | |
