# Supplementary material for: Restoring trust in truth-seekers: Effects of op/eds defending journalism and justice
Source: PLoS One. 2021 May 21;16(5):e0251284. doi: 10.1371/journal.pone.0251284 (PMC8139466; doi:10.1371/journal.pone.0251284)
Supplement: S2 Appendix — (DOCX) [file pone.0251284.s002.docx]

**S2 Appendix. Study 2 Stimulus Video Transcripts.**

*Treatment: Defense of Professionalism*

[BLACK SCREEN WITH CAPTION: Video 1 of 2 – Fox News. Shepard Smith, Fox News Chief Anchor.]

*Shephard Smith:*

For those of you at home, if I may, journalists are not the enemies of the people. It’s quite the opposite. Our profession is enshrined in the Constitution and the Fourth Estate holds the essential job of being your eyes and often ears and, when appropriate, your voice. It’s a cornerstone of our Republic. Over 22 years at this network, it has been my experience that the journalists of Fox News and Fox Business and those of our colleagues and competitors at CNN and MSNBC, NBC, ABC, and CBS, Bloomberg, PBS, Wall Street Journal, the New York Post, the New York Times, the Washington Post,… all of them operate in the public interest. We work to discern what is truth, then to present it to you in context and with perspective. And as your representatives in the people’s halls, report to you without fear or favor while striving to hold those in power to the same standards. This is the foundation of what we do. It’s our reason for being, our purpose.

[BLACK SCREEN WITH CAPTION: Video 2 of 2 – PBS NewsHour. David Brooks, New York Times Columnist]

*Judy Woodruff:*

Could this campaign, this effort by some Republicans in the House and with support from the White House to undermine the FBI, could that have a long-lasting effect on the Justice Department in the end?

*David Brooks:*

Yes, I think so. One of the things that people should know is that there are honest brokers in Washington. There are career people who really do their job, and they try to be good umpires. And some of those people, by the way, have private political opinions, but they leave that at the door when they go to work. And the FBI is filled with honest brokers, the Congressional Budget Office. There are a lot of agencies that are filled with honest brokers, and the idea that everybody in this city is a politician is just not true. It's always amazing to me that a lot of people in government, they are not actually that political. They believe in the public service and they try to do their jobs, but they're not sort of super political people. They just believe in public service. But there's been a campaign to say, no, those doesn't exist, it's all politics, everybody is partisan.

*Control: Immigration*

[BLACK SCREEN WITH CAPTION: Video 1 of 2 – Fox News. Shepard Smith, Fox News Chief Anchor.]

*Shephard Smith:*

The U.S. will deploy some 5,000 troops to the Southern border as early tomorrow as thousands of migrants make their way North from Mexico. Tomorrow. The migrants, according to Fox News reporting, are more than two months away. If any of them actually come here. But tomorrow is one week before the midterm election, which is what all of this is about. There is no invasion. No one is coming to get you. There is nothing at all to worry about. When they did this to us, got us all riled up in April, remember. The result was 14 arrests. We’re America. We can handle it. But, like I said, a week to the election.

[BLACK SCREEN WITH CAPTION: Video 2 of 2 – PBS NewsHour. David Brooks, New York Times Columnist]

*Judy Woodruff:*

For some analysis on that and more, it is Shields and Brooks time, that is syndicated columnist Mark Shields and New York Times’ columnist David Brooks. Hello to both of you.

So… David, the President, we’ve been talking about it all week, out on the trail, everyday in a way presenting a darker and darker picture of what’s gonna happen if the migrants come across the border, if we let too many immigrants in, if this happens, or that happens. Is this a successful strategy from his part?

*David Brooks:*

Yeah, releasing a pretty straight-up racist video-ad, so… it’s a surprising strategy. The guy is sitting on the best economy of our lifetime, potentially. Wages are going up even for those at the bottom of the skillset scale, so he could have a very good story to tell: hey you may not like me, but I gave you this economy. But that is not what he’s doing. He mentions the economy, of course, but he’s closing on immigration. And it’s a big risk… and it’s basically telling a lot of people that this is the heart and soul of the Republican Party, building a wall, keeping immigrants out. And it’s a message built quite on a lot of bigotry.
